# BCL::EMAS — Enantioselective Molecular Asymmetry Descriptor for 3D-QSAR

**DOI:** 10.3390/molecules17089971

**Published:** 2012-08-20

**Authors:** Gregory Sliwoski, Edward W. Lowe, Mariusz Butkiewicz, Jens Meiler

**Affiliations:** Department of Chemistry, Pharmacology, and Biomedical Informatics, Center for Structural Biology, Institute of Chemical Biology, Vanderbilt University, Nashville, TN 37232-6600, USA

**Keywords:** QSAR, CADD, stereochemistry, enantiomer, molecular asymmetry, chirality

## Abstract

Stereochemistry is an important determinant of a molecule’s biological activity. Stereoisomers can have different degrees of efficacy or even opposing effects when interacting with a target protein. Stereochemistry is a molecular property difficult to represent in 2D-QSAR as it is an inherently three-dimensional phenomenon. A major drawback of most proposed descriptors for 3D-QSAR that encode stereochemistry is that they require a heuristic for defining all stereocenters and rank-ordering its substituents. Here we propose a novel 3D-QSAR descriptor termed Enantioselective Molecular ASymmetry (EMAS) that is capable of distinguishing between enantiomers in the absence of such heuristics. The descriptor aims to measure the deviation from an overall symmetric shape of the molecule. A radial-distribution function (RDF) determines a signed volume of tetrahedrons of all triplets of atoms and the molecule center. The descriptor can be enriched with atom-centric properties such as partial charge. This descriptor showed good predictability when tested with a dataset of thirty-one steroids commonly used to benchmark stereochemistry descriptors (r^2^ = 0.89, q^2^ = 0.78). Additionally, EMAS improved enrichment of 4.38 versus 3.94 without EMAS in a simulated virtual high-throughput screening (vHTS) for inhibitors and substrates of cytochrome P450 (PUBCHEM AID891).

## 1. Introduction

Stereoisomers are defined as different molecular species of equal constitution which are separated by energy barriers [1]. For organic molecules stereochemistry is most frequently caused by carbon atoms with four different substituents. However, other stereocenters exist such as positively charged nitrogen atoms with four different substituents, double bonds with different substituents on each of the two carbon atoms, stereoisomeric allenes, atropisomeric biphenyls, *etc*. Enantiomers are a subset of stereoisomers that are defined as non-superimposable mirror images (*enantios* being Greek for opposite and *meros* for part). Despite their structural similarities, enantiomers can display very different pharmacological profiles. Stereoisomers that are not enantiomers are called diastereomers. Stereoselectivity is widely prevalent in nature as most proteins are formed from the genetically encoded L-amino acids making small molecule binding pockets enantioselective [2]. In drug discovery, there are examples in which different enantiomers show different efficacies, e.g., dexrabeprazole [3] and beta blockers [4], and different toxicities, e.g., levobupivacaine [5]. In 1992, the FDA issued a statement requiring that the development of any racemate (mixture of a compound’s stereoisomers) carry a justification for the inclusion of both isomers [6] and in the year 2000, chiral drugs accounted for over $100 billion in sales [7]. Between 1985 and 2004, the number of single enantiomer drugs as a percentage of chiral molecules increased from 31.6% to 89.8% [8].

Given the importance of stereoselectivity in drug design, it is necessary that any computational approach to drug discovery distinguishes between stereoisomers. In Structure-Based Computer-Aided Drug Discovery (SB-CADD) stereochemistry is explicitly accounted for as the molecule is docked into a structural model of the protein binding site. The 3D structure of the molecule in complex with the protein is evaluated taking its stereochemistry into account. In complex with the target protein even enantiomers turn into diastereomers and can be distinguished. In Ligand-Based Computer-Aided Drug Discovery (LB-CADD) the chemical structures of active compounds are compared to derive common features that determine activity. The task of distinguishing stereoisomers and in particular enantiomers becomes more challenging as stereochemistry needs to be defined in the absence of the protein. This is impossible in 2D molecular descriptors where only the constitution of a molecule is taken into account. Therefore extensions to 2D molecular descriptors have been developed—sometimes described as 2.5D descriptors—that describe configuration and can therefore define stereochemistry. Lastly, 3D descriptors based on the molecular conformation can define stereochemistry, if appropriately designed.

The IUPAC convention for distinguishing stereoisomers is the Cahn-Ingold-Prelog (CIP) convention distinguishing *R* (*rectus*) and *S* (*sinister*) configuration of stereocenters. It requires a priority weighting system for the different substituents that is incapable of dealing with some complex scenarios. Extensions to the CIP system have been introduced to handle situations in which the chiral center did not rest on an atom (chirality plane or axis) and for stereoisomers which do not possess centers of chirality at all (stereisomeric allenes, atropisomeric biphenyls, and ansa-compounds) [1]. Further complications arise for pseudoasymmetric stereogenic units, defined as pairs of enantiomorphic ligands together with two ligands which are non-enantiomorphic. In cases such as these, the priorities of two substituents depend on their own chiral centers. One particular disadvantage is that the CIP nomenclature does not always follow chemical intuition. For example, take the two molecules HC(CH_3_)(OH)F and HC(CH_3_)(SH)F. Naively we would align these close derivatives by superimposing H with H, CH_3_ with CH_3_, OH with SH and F with F. This assigns *R*-HC(CH_3_)(OH)F to *S*-HC(CH_3_)(SH)F and *vice versa*. In fact, closely related derivatives that place similar functional groups in the same regions of space and are likely to have similar activity can have opposite CIP assignment. Therefore, the CIP convention is not suitable to describe stereochemistry effectively for LB-CADD. 

Extensions to 2D-QSAR have been proposed to distinguish enantiomers. Golbraikh and co-workers introduced a series of chirality descriptors that use an additional term called the chirality correction added to the vertex degrees of asymmetric atoms in a molecular graph [9]. This method is similar to one proposed by Yang and Zhong [10] where the chiral index was instead appended to the substituents attached to the chiral center. Multiple similar algorithms have also been proposed [11,12,13,14]. For example, Brown, *et al.* [11] added chirality to their graph kernel method. The drawbacks of these methods include their reliance on the problematic R/S designations as well as the combination of spatial and atom property information such that their indices become a principally mathematical concept with little interpretation on physical terms.

Another approach proposed by Benigni and co-workers [15] describes a chirality measure based on the comparison of the 3D structure for a molecule with all others in a data set. Zabrodsky [16] proposed a similar continuous symmetry measure which quantifies the minimal distance movement for points of an object in order to transform it into a shape of desired symmetry. However, these molecular similarity indices are very sensitive to relative orientation and depend on pairwise molecular indices which can complicate QSAR-based high throughput screening.

Aires-de-Sousa, *et al.* [17,18,19] introduced a 3D-QSAR method for handling enantiomers. Classical 3D-QSAR descriptors such as radial distribution functions are incapable of distinguishing between enantiomers based on their nature. This method employs an RDF-like function that utilizes a ranking system for each chiral center introduced by Zhang and Aires-de-Sousa that reinterpreted the CIP rules in terms of more meaningful physicochemical properties. Additionally, it has the benefit of being a vector rather than single value which is equal and opposite for enantiomer pairs. However, this method requires the identification and appropriate labeling of all stereogenic units and suffers from the fact that spatial information is combined with atom properties where some physical interpretability is lost. It is also worth mentioning that it is not clear if it is pharmacologically relevant to specify every stereogenic component of a molecule, but rather if different profiles between enantiomers depend on specific chiral centers and/or an overall chirality of the molecule as a whole.

CoMFA [20] is an appealing method for distinguishing between enantiomers as it avoids the necessity to identify stereogenic centers. Rather, it intrinsically takes chirality into account as the molecular fields of chiral isomers are inherently different. However, the method relies on superimposition of all molecules [9] which is difficult to achieve for large or diverse substance libraries.

Here we propose a novel enantioselective 3D descriptor for QSAR that is similar to the RDF-like function proposed by Aires-de-Sousa and co-workers but with important differences to address the concerns raised above. We call this new method Enantioselective Molecular ASymmetry (EMAS). Our method does not rely on any priority ranking or distinction of every stereogenic unit, thereby eliminating the need to combine spatial and atomic properties and bypassing the difficulties that arise in non-conventional chiral centers. Rather, the enantiomeric distinctions “emerge” from the spatial distribution of atoms within the molecule. Additionally, EMAS is designed to avoid a rigid distinction between enantiomers but rather to represent the overall asymmetry of a molecule as it compares to other similar molecules as well as its own enantiomorphs. Therefore, EMAS intends to describe overall molecular asymmetry while including a directionality component that can distinguish between enantiomers.

## 2. Results and Discussion

### 2.1. Enatiomorphism is Determined by Asymmetry in Shape or Property Distribution

Enantiomorphism in small molecules is impacted by two phenomena. The first factor is the shape of the molecule—*i.e*., the distribution of its atom coordinates in space. If the mirror image of this shape cannot be superimposed with the original version, the two molecules are enantiomers. Beyond the overall shape the distribution of properties plays a role. We can envision molecules that have a (near) perfect symmetric shape. Image and mirror image will be identical shape wise. However, distribution of partial charge, polarizability, and electronegativity can be enantiomorphic. While both contributions are coupled they represent two dimensions of one phenomenon. For a specific molecule one of the other factors might be more pronounced. For example steroids can have enantiomorph shapes but have relatively uniform property distributions as they are dominated by apolar CH groups. On the other hand, the molecule CFClBrI is an almost perfect regular tetrahedron with a highly enantiomorph distribution of partial charge and polarizability. As both contributions can determine properties and activities of small molecules, stereochemical descriptors should capture and ideally distinguish both contributions.

### 2.2. Radial Distribution Functions Separate Shape Information and Property Distribution

Radial Distribution Functions (RDFs) are often applied in 3D-QSAR [21,22]. As a means of comparison, the general form of the atomic radial distribution function is shown:


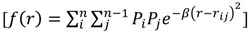
(1)

In this equation, 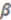 is a smoothing parameter, often called the ‘temperature’ while 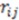
is the distance between atoms 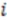
and 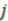, 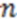
is the total number of atoms in the molecule, and 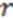 is the running variable for the function 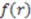. Often, such equations are ‘weighted’ with a property coefficient for both atoms 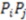. The function plots shape (*i.e*., distance between two atoms) on the x-axis, the respective property coefficient on the y-axis thereby separating geometry from property distribution. With 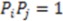
this function is a representation of the overall shape of the molecule based on the frequencies of all atom pair distances within each radial distance step. As distances are invariant to mirroring, enantiomers share identical RDF functions. Note that diastereomers have distinct RDFs as not all atom pair distances are identical. 

### 2.3. Expanding RDFs to ‘Signed’ Volumes that Are Sensitive to Shape Enantiomorphy

We first look for the simplest geometric form that would be sensitive to mirroring. This shape would be a tetrahedron. We choose tetrahedrons consisting of all combinations of three atoms 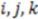 and the center of the molecule. Other approaches use all permutations of four atoms. The present approach reduces the computational demand. The geometric property plotted for the tetrahedron is volume. 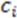, molecules-17-09971-i013, and molecules-17-09971-i014are the coordinates of the three atoms. The center of the molecule is defined by point molecules-17-09971-i015. Then, we compute the signed volume as:


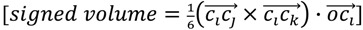
(2)

While the absolute term always reflects volume, it is important to note that the result can have a positive or negative sign, depending on the order of points which is initially arbitrary. We note that the volume has an arbitrary sign that inverts when the molecule is converted into its mirror image. We note further that the volume becomes 0 if the plane defined by 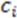, 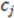, and 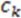 includes 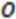. This property is beneficial as a planar arrangement of atoms cannot be enantiomorphic. However, for a tetrahedron to contribute to enantiomorphy, its edges 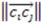, 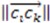, and 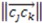 must be of different length. This property is captured by a stereochemistry score:


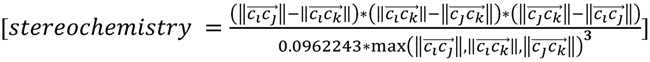
(3)

**Figure 1:**
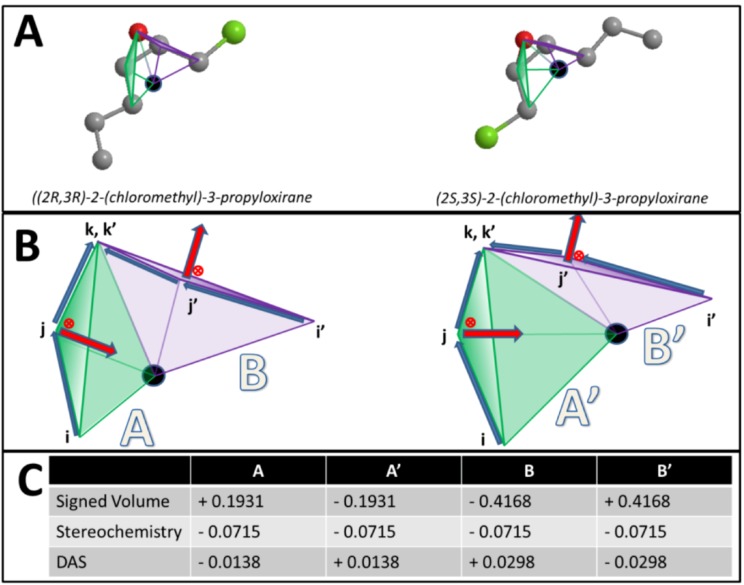
Calculating DAS (**A**) Scores reflect opposing enantiomorphs based on cross-product direction and geometric center. Enantiomers [(2*R*,3*R*)-2-(chloromethyl)-3-propyloxirane and (2*S*,3*S*)-2-(chloromethyl)-3-propyloxirane] with two stereocenters are shown. (**B**) Two triangles are visualized in both enantiomers. These triangles encompass the same triplets of atoms between the two molecules. Four tetramers formed by the atom triplets and molecular center are visualized. i, j, k, and i', j', k' reflect the order of these atoms in either molecule. Importance of atom ordering is shown based on the direction of cross product (red arrow) and location of molecular center (black circle). (**C**) Volume and score calculations for the four tetrahedrons across both enantiomers are shown. Note the opposite signs and scores between the two enantiomers’ tetrahedrons.

**Figure 2:**
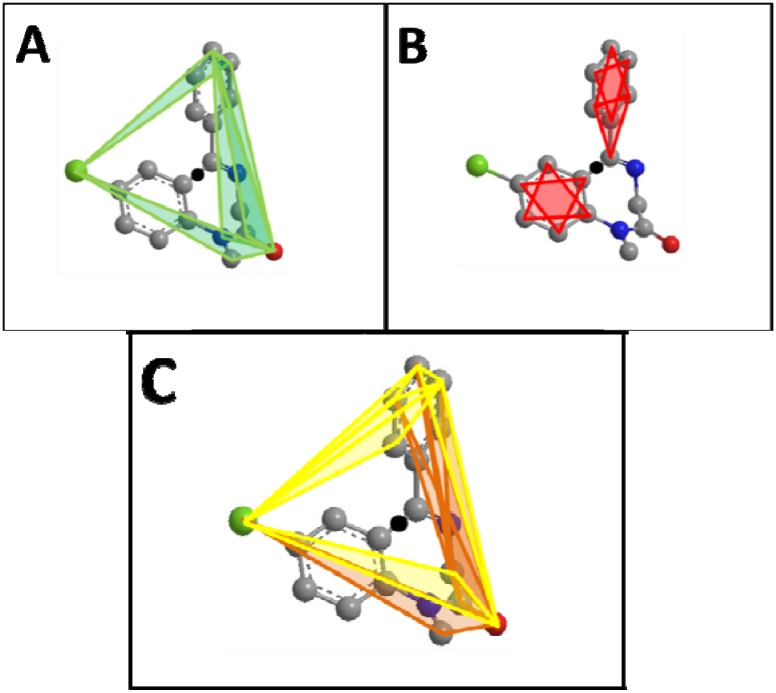
Diazepam (**A**) Top five scoring atom triplets in diazepam are shown. The black circle in all figures represents the molecular center. (**B**) Lowest five scoring atom triplets in diazepam. All triplets shown here score 0 and do not contribute to the RDF-like code. (**C**) Top five positive and top five negative scoring triplets in diazepam. Here is visualized the different distribution of high scoring positive (yellow) versus high scoring negative (orange) triplets in diazepam.

Two things emerge from the numerator: the asymmetry is evaluated based on the variation in distances between the three atoms. If any two distances are equal, the triangle formed from the three atom coordinates will contain perfect symmetry and the score will be 0. Additionally, the directional (enantiomorphic) information emerges based on the order of distances. For example, if 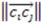 > 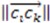 > 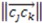, then this product will have a negative sign 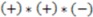. However, if, from the vantage point of the molecular center, the order of distances has been shuffled (as would be seen in an enantiomer 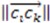 > 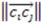 > 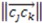), the sign changes as well 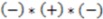. Figure 1 demonstrates how opposite directions emerge depending on the ordering of instances. Recall that by allowing a signed volume, we ensure that the order of distances does not rely on the order of atoms coordinates encountered, but rather as the order of distances seen from the molecular center in terms of the cross product’s direction. The score is normalized by a constant factor of 0.0962243 which is calculated as the maximum possible score when the largest of the three distances is 1. Details can be found in the supplemental information. Figure 2 compares atom triplets that give rise to high versus low scores as well as scores with opposite directions.

The final directional asymmetry score (DAS) of any given atom triplet becomes:


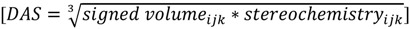
(4)

**Figure 3:**
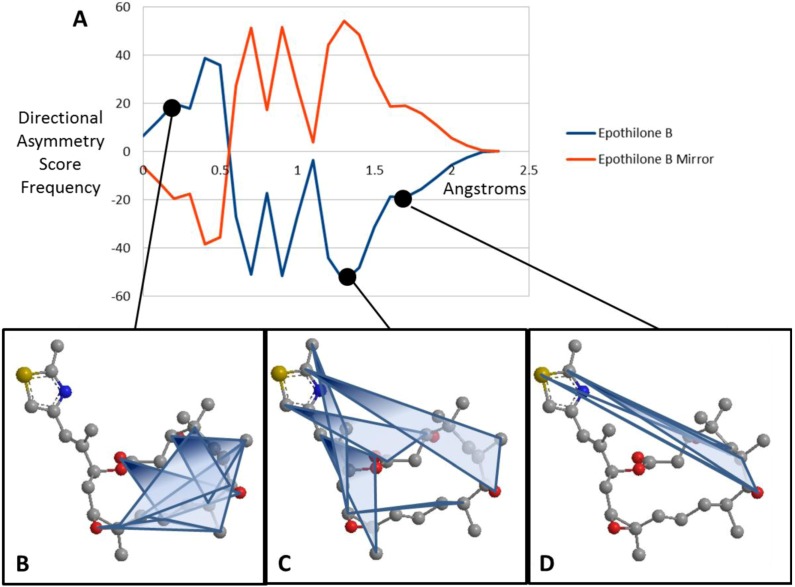
EMAS curves for epothilone B (**A**) Plotted EMAS curves for epothilone B (blue) compared with its mirror image (red). X-axis represents the Directional Asymmetry Score in angstroms while the y-axis indicates the frequency of these scores across the entire molecule. (**B**) Atom triplets with a directional asymmetry score of approximately 0.3 angstroms. Note that these triangles generally cover the center of the molecule and are fairly symmetric. (**C**) Atom triplets with a directional asymmetry score of approximately 1.3 angstroms. Note that these triangles are further from the center of the molecule and have an asymmetric shape. **(D)** Atom triplets with a directional asymmetry score of approximately 1.7 angstroms. Note that these atom triplets lie furthest from the center of the molecule and are very asymmetric.

Note that the products cube-root has been taken to achieve a dimension of distance resembling a common RDF. This procedure preserves the sign and expands the range of frequently occurring low-scoring triplets at the cost of rare triplets with high scores. Substituting this directional asymmetry in place of atom distance, the EMAS function becomes:


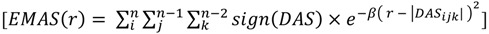
(5)

where 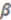 is the smoothing parameter, 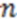
is the total number of non-hydrogen atoms, and 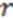
is the running variable of the function 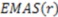. The alternate sign preceding the exponential function transfers the “directionality” of the score to the overall function so that at any given score, the intensity reflects the subtraction of negative (one direction) from positive (opposite direction). Figure 3 maps the EMAS plot for epothilone B and its mirror image.

As with the basic radial distribution function, the absence of any weighting coefficient results in a descriptor that encodes only spatial information. While this is important information in and of itself, the addition of a property weighting coefficient increases the utility of this descriptor. Since we are iterating over all atom triplets, the possibility that one atom property can throw off two other atom properties in unintended ways made it problematic in some cases to simply multiply the three atom properties together. Adding the properties, on the other hand, can circumvent this issue but two atom properties of equal magnitude and opposite signs can cancel each other out. Therefore, we retained the functionality for both property coefficient methods and suggest that any use of this descriptor in larger datasets test either method since one may outperform the other depending on the dataset.

### 2.4. Evaluation of EMAS as a Novel Descriptor

#### 2.4.1. Predictability Benchmarking: Cramer’s Steroids

A commonly used dataset for evaluating the predictive capability of novel stereochemistry-based descriptors was introduced by Cramer *et al*. in 1988 [20] and several structures were corrected in a subsequent publication [23]. These thirty-one steroid structures are accompanied with their experimental binding affinities to human corticosteroid-binding globulins (CGB) and provide a small dataset containing many stereocenters. Additionally, the rigidity of these compounds makes them an ideal benchmark set for 3D-QSAR algorithms eliminating the factor of conformational flexibility. Since EMAS can be employed in three forms: spatial only, property weighting coefficient via summation, and property weighting coefficient via multiplication, we trained three separate artificial neural network (ANN) models using descriptors derived in each of these three methods. To predict binding affinities over the entire dataset, we used a cross-validated leave-one-out approach. To compare the predictive power of our model versus other descriptors that have been tested against the steroid set, we calculated the correlation coefficient 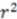 of predicted versus experimental affinities and the “cross-validated 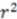
” 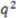. 

As expected, the ANN model generated using no property weighting (solely spatial information) performed the worst of the three, producing a 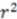 of 0.78 and a 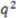 of 0.60. By weighting with a multiplicative property coefficient, the performance increased considerably, resulting in a 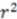 of 0.86 and a 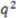 of 0.74. Weighting with the property summation coefficient yielded the best predictions with a 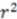 of 0.89 and a 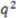 of 0.78. 

Since we began with an interest in generating a molecular asymmetry descriptor that could distinguish between enantiomers, we wanted to ensure that the inclusion of directionality increased the information contained in the descriptor. Therefore, we created a version of the descriptor that incorporates just the absolute value of all stereochemistry scores, thereby eliminating all directional information while retaining all other spatial information. We found that by training our model without directional information, the predictive capabilities for the steroid affinities decreased to a 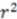 of 0.65 and a 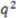 of 0.41, reinforcing our original design to capture stereochemistry. We also compared the model employing EMAS with one created with a traditional RDF. This model performed worse than any of our three methods giving a 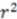 of 0.75 and a 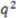 of 0.56. Weighting the RDF’s with the same properties used to weight EMAS did not produce any significant improvement in the model (data not shown). Cross-validated predictions for all variations of EMAS as well as the experimental affinities can be found in Table 1.

**Table 1:** Experimental and predicted binding affinities for the 31 Cramer’s steroids using novel stereoselective descriptor to train ANN models. Spatial predictions utilize the novel descriptor without any atom property weighting. Multiply properties utilize the novel descriptor weighted by the product of atom properties. Sum properties utilize the novel descriptor weighted by the sum of atom properties.

Molecule	Observed CBG affinity (pKa)	Predicted [spatial]	Predicted [multiply properties]	Predicted [sum properties]	Predicted [no stereochemistry]
aldosterone	−6.28	−7.47	−7.31	−7.25	−7.22
androstanediol	−5.00	−5.47	−5.46	−5.33	−5.56
5-androstenediol	−5.00	−5.47	−5.43	−5.36	−5.75
4-androstenedione	−5.76	−5.64	−5.60	−5.79	−6.36
androsterone	−5.61	−5.78	−5.81	−5.55	−5.42
corticosterone	−7.88	−7.30	−7.37	−7.32	−7.34
cortisol	−7.88	−7.63	−7.58	−7.64	−7.33
cortisone	−6.89	−7.22	−6.83	−7.39	−7.07
dehydroepiandrosterone	−5.00	−5.39	−5.13	−5.46	−5.80
11-deoxycorticosterone	−7.65	−7.48	−7.47	−7.50	−6.85
11-deoxycortisol	−7.88	−7.66	−7.53	−7.59	−7.52
dihydrotestosterone	−5.92	−5.38	−5.70	−5.43	−5.96
estradiol	−5.00	−5.40	−5.36	−5.32	−5.21
estriol	−5.00	−5.25	−5.26	−5.43	−6.10
estrone	−5.00	−5.30	−5.21	−5.54	−5.42
etiocholanolone	−5.23	−6.42	−6.44	−6.22	−6.27
pregnenolone	−5.23	−5.30	−5.25	−5.37	−6.37
17a-hydroxypregnenolone	−5.00	−5.20	−5.28	−5.29	−6.65
progesterone	−7.38	−7.17	−7.27	−7.13	−6.46
17a-hydroxyprogesterone	−7.74	−7.42	−7.39	−6.97	−6.70
testosterone	−6.72	−6.08	−6.36	−6.19	−5.94
prednisolone	−7.51	−7.61	−7.36	−7.65	−7.03
cortisolacetat	−7.55	−6.74	−6.90	−7.63	−6.00
4-pregnene-3,11,20-trione	−6.78	−6.40	−6.83	−6.09	−6.46
epicorticosterone	−7.20	−5.98	−6.00	−7.03	−7.15
19-nortestosterone	−6.14	−5.58	−5.86	−5.54	−5.45
16a,17a-dihydroxy-progesterone	−6.25	−7.25	−7.04	−7.46	−7.36
16a-methylprogesterone	−7.12	−6.69	−6.39	−6.78	−6.60
19-norprogesterone	−6.82	−6.01	−6.30	−7.25	−6.19
2a-methylcortisol	−7.69	−6.62	−7.22	−7.68	−6.57
2a-methyl-9a-fluorocortisol	−5.80	−7.56	−6.97	−6.22	−6.74
	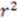	0.78	0.86	0.89	0.65
	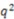	0.60	0.74	0.78	0.42

Since this dataset is well-established across similar descriptors in the literature, we compared our predictive power to other methods and found that our best 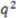 fell at the average 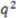 of all of these methods (0.63 < 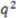 < 0.94). This result is somewhat difficult to interpret for several reasons: (a) different statistical models are utilized; (b) different degrees of cross validation were employed, and (c) our descriptor solely describes stereochemistry and is meant to be complemented by other descriptors (read below). Most of the competing descriptors include more information on molecule size, shape, and property distribution. However, it is important to note that while EMAS does not require any molecular alignment or pre-annotated stereocenters, it is capable of performing well with a dataset that contains a great deal of stereochemistry. Additionally, the inclusion of directional information outperforms a similar implementation lacking directional information as well as the similar RDF descriptor weighted with or without atom properties. For a comparison of our 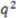 with other documented tests against Cramer’s steroids, see Table 2.

**Table 2:** Comparison of novel stereoselective descriptor predictability with other published QSAR methods against the Cramer’s steroid set. Calculation of 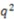 can be found in the methods section. Statistical model generation method is indicated as well as QSAR method employed are indicated for each reference.

QSAR Method	Model Creation	q^2^	Reference
**Purely Spatial RDF-like stereochemistry**	Artificial Neural Network	**0.56**	
**Property weight RDF-like stereochemistry (product)**	Artificial Neural Network	**0.74**	
**Property weight RDF-like stereochemistry (sum)**	Artificial Neural Network	**0.78**	
Stochastic 3D-chiral linear indices	Multiple Linear Regression	0.87	[13]
Chiral Topological Indices	Stepwise Regression Analysis	0.85	[10]
Chiral Graph Kernels	Support Vector Machine	0.78	[11]
Chirality Correction and Topological Descriptors	K-nearest neighbor	0.83	[9]
Molecular Quantum Similarity Measures	Multilinear Regression	0.84	[24]
Shape and Electrostatic Similarity Matrixes	Non-linear Neural Network	0.94	[25]
Comparative Molecular Moment Analysis	Partial Least Squares (PLS)	0.83	[25]
Comparative Molecular Similarity Indices Analysis	PLS	0.67	[26]
Comparative Molecular Field Analysis	PLS	0.65	[20]
E-state Descriptors	PLS	0.62	[27]
Molecular Electronegativity Distance Vector	Genetic Algorithm PLS	0.78	[28]
Molecular Quantum Similarity Measures	Multilinear Regression and PLS	0.80	[29]

#### 2.4.2. vHTS Utility and Enrichment Benchmarking: PUBMED AID891

We provide the above analysis for comparison. However, realistically the steroid dataset is too small to provide a good benchmark for EMAS as often the number of features (24 features) is in the same order of magnitude as the number of data points (31 molecules). Therefore we tested the descriptor in a virtual high-throughput screening (vHTS) endeavor. For the benchmark dataset, we used the publicly available results of a conformational screen for inhibitors and substrates of cytochrome P450 2D6 (AID 891). This dataset is of moderate size (approximately 10,000 molecules) and contains both active (18%) and inactive (82%) compounds. We employed a forward-feature selection (FFS) analysis that selects optimal descriptors from RDF’s, 3D Autocorrelations (3DA), and 2D Autocorrelations (2DA) functions labeled with atom properties including charge, electronegativity, and effective polarizability (see Experimental section). For a complete list of features tested in forward-feature selections, please see supplemental Table S1. ANN 3D-QSAR models were trained with and without inclusion of the EMAS descriptors in the list of descriptors for FFS to choose from. Hence the utility of the EMAS descriptor can be evaluated in two ways: (a) are the EMAS descriptors chosen by the FFS procedure? and (b) has the final model that includes EMAS descriptors an increased predictive power? The FFS with the default set of initial features resulted in a best descriptor set of 9 features distributed evenly across RDF’s, 3D Autocorrelations (3DA), and 2D Autocorrelations (2DA). Cross-validated predictions from the ANN model constructed with this feature set produced an enrichment of 3.94 and a receiver operating characteristic (ROC) curve with an area under the curve (AUC) of 0.826. 

**Figure 4:**
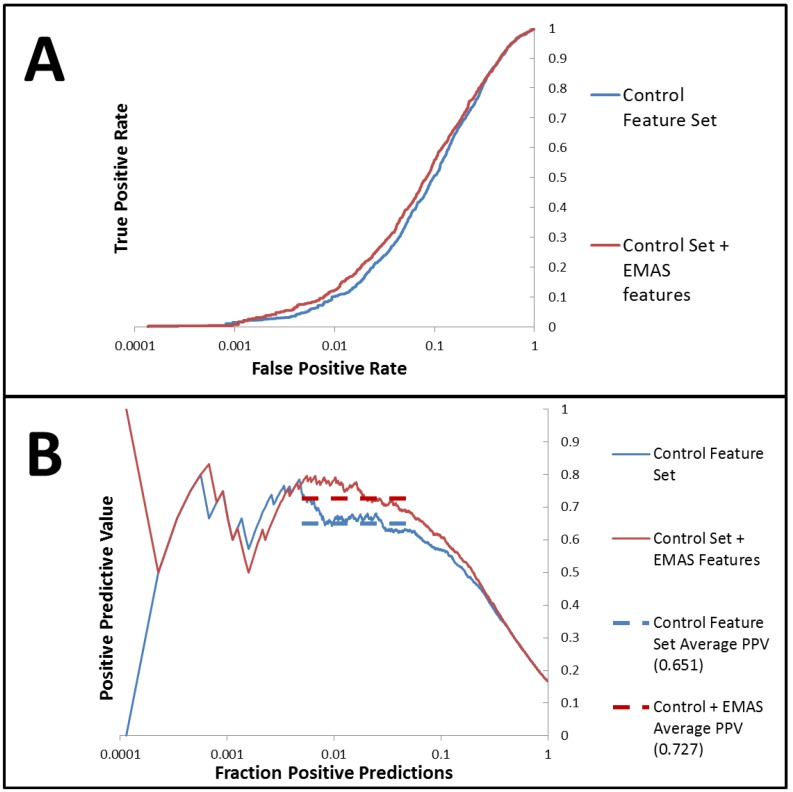
ROC and PPV results for the feature forward analysis with the control set of features compared with the control set combined with EMAS features (**A**) AID891 prediction ROC curves generated from the ANN models trained with the best descriptor set generated from the forward feature analysis beginning with the control set of features combined with the novel EMAS features (red) show improved performance when compared with ROC curves generated from the ANN models trained with the best descriptor set generated from the forward feature analysis beginning with the control set of features (blue) (**B**) PPV curves for models trained with the best descriptor set of control features combined with the EMAS features (red) shows improved performance over those models trained with the best descriptor set of control features only (blue). Dashed lines of corresponding colors show the average PPV values over the FPP region from which the models were optimized (0.005 to 0.05 fraction positive predicted values).

An identical FFS analysis was performed by combining the default set of features with 34 EMAS features including all three variations of EMAS (spatial, property weighting via sum, and property weighting via product) weighted with the same list of properties used to test RDFs, 3DAs, and 2DAs. The best set of features contained 20 total features distributed across RDF’s, 3DA’s, 2DA’s, number of hydrogen bond donors, and several EMAS features. There were a total of seven EMAS features represented in the best feature set. Therefore, almost one third of the total features in the best feature set generated through this analysis were EMAS features. This set of seven features contained a spatial EMAS weighted by van der Waals surface areas, three EMAS features weighted via the product method and three EMAS features weighted via the sum method. This substantial representation of EMAS in the best feature set suggests that EMAS successfully provides useful information for the model development that may not be represented in any other feature in the original set. Cross-validated predictions from the ANN model constructed from this EMAS-inclusive feature set produced an enrichment of 4.38 and a ROC curve with an area under the curve of 0.837. Positive predictive value (PPV) is a related measure of a model’s predictive capability which tracks predictive precision as more and more positive predictions are made. By comparing the average PPV precision over a range of the fraction of total predictions made (fraction positive predictions, FPP) of interest, it is possible to compare predictive capabilities for two models. Over the FPP range of 0.005 to 0.05, we find that our model trained with the EMAS features performed significantly better than the model trained without EMAS features (0.727 PPV precision compared with 0.651). A paired t-test for the cross-validated models comparing precisions in this FPP range showed that this is a statistically significant improvement (*p* < 0.005) over the analysis completed without EMAS features. For a complete list of the best features determined from both forward feature analyses, please see the supplemental Table S2. Comparative ROC and PPV curves from the forward feature analyses for the control set of features and the control set combined with EMAS features are shown in Figure 4.

## 3. Experimental

### 3.1. Generation of Numerical Descriptors for QSAR Model Creation

3D models of all small molecules were generated using the CORINA software package unless already defined. For feature selection analysis, a set of 2,100 numerical descriptors was generated using the BioChemical Library (BCL) software created in our lab. The descriptors can be classified into five categories, including six scalar descriptors (molecular weight, number of hydrogen bond donors, number of hydrogen bond acceptors, logP, total charge, and topological surface area), 18 2-dimensional auto-correlation functions, 18 3-dimensional autocorrelation functions, 18 radial distribution functions, and 34 novel molecular asymmetry descriptors. These 34 descriptors included spatially-based asymmetry functions with and without van der Waals (VDW) surface area scaling, 16 property-weighted asymmetry functions based on the multiplicative scheme, and 16 property-weighted asymmetry functions based on the additive scheme. These properties included sigma charge [30,31,32], pi charge [33,34,35], Vcharge [36], total charge [30,31,32,33,34,35], sigma electronegativity [30,31,32], pi electronegativity [33,34,35], effective polarizability [37,38,39], and lone pair electronegativity [33,34,35] with and without VDW surface area scaling. The control comparison forward feature selection analysis was performed with a feature set that included all features listed above except the novel stereochemistry features. This feature set contains 1,284 features. For steroid binding predictions, descriptor sets were created using only one novel stereochemistry method and those including property weighted used the same properties listed.

### 3.2. Training, Monitoring, and Independent Dataset Generation

#### 3.2.1. Cramer’s Steroids

The dataset was split for ANN training into three subsets: training, monitoring, and independent. The monitoring dataset is necessary to prevent over-training. Because of the small size of the dataset, only one molecule was labeled independent. Five molecules were used as the monitoring dataset, 25 for training. The set of five molecules was incremented through the entire dataset for a total of 6 different monitoring sets. Leave-one-out cross validation was performed where each molecule was used as the independent molecule while the remaining 30 molecules were used for training and monitoring. The predictions were averaged across the different monitoring sets to yield the final activity predictions for the entire set of 31 molecules.

#### 3.2.2. PUBMED AID891

AID891 is a publically available dataset that can be found at http://pubchem.ncbi.nlm.nih.gov/. It contains 1,623 active compounds and 7,756 inactive compounds tested for inhibition of cytochrome P450 2D6. This dataset was split into 10 clusters distributed into a training set of eight clusters, a monitoring set of one cluster, and an independent set of one cluster. For cross validation, the monitoring and independent datasets are iterated and then the resulting independent predictions are averaged to give the final list of predicted activities that spans the entire dataset. In order to maximize model performance, the dataset was balanced through oversampling. In other words, the active compounds were represented multiple times so that the number of active compounds roughly equals the number of inactive compounds. This method of balancing has been used to maximize QSAR models in other datasets where the number of active compounds is significantly less than the number of inactive compounds [40]. 

The pIC_50_ values of each compound within AID891 and the steroid binding data for the Cramer dataset were used as output for the ANN models. For the AID891 dataset, inactive compounds were set to a pIC_50_ value of 3. The root-mean-square deviation (RMSD) between predicted and experimental activities was used as the objective function for training the ANN.

### 3.3. Artifical Neural Network (ANN) Architecture and Training

For the AID891 dataset, the ANN was trained using back propagation and a sigmoid transfer function with a simple weight update of eta = 0.1 and alpha = 0.5. The hidden layer contained eight neurons. For the steroid dataset, the ANN was trained using the same protocol as the AID891 dataset but the number of hidden neurons was reduced to 4 due to the smaller size of the dataset.

### 3.4. Forward-Feature Selection for Optimal Descriptor Set Selection

Descriptor selection was performed to test the novel descriptor against all other implemented descriptors to see if it provided an increase to enrichment over any of the other descriptors. The approach begins with a single descriptor, trains a model with only that descriptor, and then continuously adds more descriptors one at a time, training a new model each round. At the completion of each round, the descriptor set that produced the lowest RMSD score was retained for the next round. All descriptors not present in the retained list of descriptors are then added individually to that retained list of descriptors and the descriptor set producing the best RMSD score is retained for the next round, and so on. At the completion of these iterations, the round that produced the best RMSD score overall is recalled as the top descriptor set. If a descriptor appears in this list of best descriptors, then it suggests that significant information had been gleaned from that descriptor during the ANN training. 

### 3.5. Model Evaluation

ANN models using the AID891 datasets were analyzed using receiver operation characteristic (ROC) curves to assess their predictive power. These curves plot the rate of true positives versus the rate of false positives as a fraction of the total number of positives. Therefore, a slope of 1 would reflect random guesses as each true positive would be statistically likely to be followed by a false positive. An increase in slope and area under the curve would indicate an increase in predictive power. The initial section of the ROC curve is often most important because it represents compounds with the highest predicted activity. Therefore, enrichment values are determined based on the slope of the ROC curve comprising the first subset of molecules. Increases in enrichment is often the most important measure for application of virtual screening in drug discovery as it reflects the expected factor at which the fraction of actives will be increased over an unbiased dataset.

Positive predictive value (PPV) is a measure related to enrichment which tracks the model’s predictive precision as the fraction of predicted positives (FPP) increases from highest predicted activity to lowest. A model is likely to become less precise as the predicted activities approach the cutoff point and therefore it is common to specify a range of FPP of interest when measuring a PPV. FPP is calculated as the number of true positive predictions plus the number of false positive predictions divided by the size of the dataset. PPV is calculated as the number of true positive predictions divided by the total number of positive predictions (true and false positive).

To determine the statistical significance for the average PPV improvement over the FPP range of 0.005 to 0.05, we compared the average PPV within this FPP range for each combination of training and modeling datasets that went into the cross-validated model. By aligning these datasets between the two models, we were able to perform a two-tailed paired t-test to show a significant improvement for the cross validated model including EMAS features over the cross-validated model without EMAS features. 

To evaluate the utility of models trained with the steroid dataset in a way which could be comparable with published methods, the conventional correlation coefficient 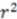 of the predicted activities against actual activities and cross validated 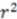, also known as 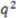 were calculated for each descriptor set. All predicted values used in these analyses were the average predicted activities from each of the leave-one-out models with the different monitoring datasets. The 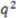 is calculated from the equation: 


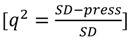
(6)

Here, 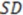 is the sum of squared deviations of each biological property from their mean and 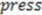 (predictive residual sum of squares) is the sum of the squared differences between the actual biological property and the cross-validated predicted property.

### 3.6. Implementation

The descriptor generation and ANN algorithms were implemented in the BioChemistryLibrary (BCL) version 2.4. The BCL is a C++ library that includes classes to model small molecules as well as larger molecules such as proteins. It contains force-fields, optimization algorithms, and different prediction approaches such as neural networks and support vector machines to model molecular structures, interactions, and properties. This application will be made freely available for academic use at http://www.meilerlab.org/. The training method used is simple propagation, a supervised learning approach. All C++ ANN trainings were performed on a Dell T3500 workstation equipped with 12GB RAM and an Intel(R) Xeon(R) W3570@3.20GHz running 64-bit CentOS 5.2. 

## 4. Conclusions

The goal of this project was to develop a 3D-QSAR descriptor that was capable of not only distinguishing between enantiomers, but also of describing the overall degree of asymmetry for a molecule. This was accomplished by developing an RDF-like curve that described the distribution of ‘directional asymmetry scores (DAS)’ rather than inter-atomic distances. The DAS is designed to incorporate information regarding the degree and direction of asymmetry between each atom triplet in the molecule. The degree of asymmetry is calculated as a product of how asymmetrically the three atoms are distributed and the distance they lie from the center of the molecule. This asymmetry is related to the differences between their interatomic distances and the distance from the center of the molecule is related to the volume of the tetrahedron created by the three atom coordinates and the geometric center of the molecule. The direction of asymmetry is related to the distribution of the interatomic distances between these three atom coordinates from the point of view of the center of the molecule. If the sides of the triangle created by these three atoms are different, then identical triangles “pointing” in opposite directions will have a different ordering of sides depending on which direction they “point.” This is the key variable that allows the descriptor to distinguish between enantiomers. To exclude any influence that the order in which atoms are listed in the molecule may play on this directionality scheme, we offset this by incorporating the cross product of the two vectors created from the three atoms. This cross product will swap signs when the atoms are ordered differently thereby eliminating the influence of the order of atoms.

We tested the value of this descriptor by training ANN 3D-QSAR models. In order to provide a basis of comparison with other documented QSAR methods that address stereoselectivity, we used a small dataset of steroids that is commonly used as a benchmark for these types of descriptors. We found that the predictability of our descriptor performed comparably with other stereochemistry-based descriptors when evaluated with this set of 31 steroids (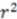 = 0.89, 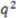 = 0.78). Additionally, we assessed the utility of the EMAS descriptor by running vHTS experiment on a publically available dataset (PUBCHEM AID 891). A forward-feature selection analysis that determines the most effective set of descriptors for this dataset was employed and the best set of features included several EMAS functions (seven EMAS of 20 total features). This set of features improved the performance of our models over those that were tested without EMAS functions (enrichment of 4.38 when including EMAS versus enrichment of 3.94 without EMAS). 

Although our descriptor performs well with the datasets tested, it is still outperformed by several techniques with the steroid dataset. One difficulty with this dataset is that its small size adds significant noise to the results. Additionally, the cross-validation methods used to analyze the performance of these methods vary and are often more forgiving than ours. Future development of EMAS, however, can provide superior predictions even with smaller datasets and extensions to the current implementation of EMAS are being pursued in our lab. Molecular flexibility is one major avenue in which we are improving our implementation. By design, EMAS currently considers single, static conformations when scoring molecules and this may fail to incorporate widely different conformations seen in highly flexible molecules.

We conclude that the EMAS descriptor encodes stereochemistry thereby providing important information that is not captured in other 3D-QSAR descriptors. There are several published QSAR methods that performed better than ours in the steroid dataset but these methods often require some heuristic for describing the stereocenters within each of the molecules or aligning the 3D structures of these molecules. Our descriptor is not subject to either of these limitations and therefore can be extended to broader applications than those previously described.

## Supplementary Materials

Supplementary materials can be accessed at: http://www.mdpi.com/1420-3049/17/8/9971/s1.
