# Supplementary material for: BCL::EMAS — Enantioselective Molecular Asymmetry Descriptor for 3D-QSAR
Source: Molecules. 2012 Aug 20;17(8):9971–89. doi: 10.3390/molecules17089971 (PMC3805266; doi:10.3390/molecules17089971)
Supplement: Supplementary file 1 [file molecules-17-09971-s001.pdf]

Supplementary Materials

BCL::EMAS — Enantioselective Molecular Asymmetry Descriptor for 3D-QSAR

Gregory Sliwoski, Edward W. Lowe, Jr., Mariusz Butkiewicz and Jens Meiler *

Department of Chemistry, Pharmacology, and Biomedical Informatics, Center for Structural Biology, Institute of Chemical Biology, Vanderbilt University, Nashville, TN 37232-6600, USA

* Author to whom correspondence should be addressed; E-Mail: jens.meiler@vanderbilt.edu; Tel.: +1-615-936-5662; Fax: +1-615-936-2211.

Normalization of Stereochemistry Score

The stereochemistry score is normalized based on the maximum possible stereochemistry score which can be computed assuming $a \geq b \geq c$ and $c = a - b$:

$$f(a,b,c) = -(a-b)(b-c)(c-a)$$

$$= -a^3\left(1-\frac{b}{a}\right)\left(\frac{b}{a}-\frac{c}{a}\right)\left(\frac{c}{a}-1\right)$$

$$= a^3\left(1-\frac{b}{a}\right)\left(2\frac{b}{a}-1\right)\left(\frac{b}{a}\right)$$

with a^3 being a constant and $x := \frac{b}{a}$ we find: $f(x) = 3x^2 - x - 2x^3$.

$$\frac{\partial f}{\partial x} = 6x - 1 - 6x^2$$

$$0 = x^2 - x + \frac{1}{6}$$

$$x = \frac{1 \mp \sqrt[2]{\frac{2}{6}}}{2} \quad \rightarrow \quad x = \frac{1}{2} \mp \sqrt[2]{\frac{1}{12}}$$

$$b = 0.211328, c = 0.788675$$

$$\max\{(1-b)(b-c)(c-1)\} =$$

Table S1. Complete feature set used in feature selection analysis. Control set included all of the same features without novel EMAS functions.

	Descriptor Name	Description
**Scalar descriptors**	Weight	Molecular weight of compound
	HbondDonor	Number of hydrogen bonding acceptors derived from the sum of nitrogen and oxygen atoms in the molecule
	HBondAcceptor	Number of hydrogen bonding donors derived from the sum of N–H and O–H groups in the molecule
	TopologicalPolarSurfaceArea	Topological polar surface area in [$Å^2$] of the molecule derived from polar 2D fragments
	LogP	Octanol/water Partition coefficient calculated by atom-additive method
	TotalCharge	Sum of atomic formal charges across molecule
**Vector descriptors**	Identity	weighted by atom identities
2D Autocorrelation (11 descriptors)	SigmaCharge	weighted by σ atom charges
	PiCharge	weighted by π atom charges
3D Autocorrelation (12 descriptors)	TotalCharge	weighted by sum of σ and π charges
	SigmaEN	weighted by σ atom electronegativities
Radial Distribution Function (48 descriptors)	PiEN	weighted by π atom electronegativities
	LonePairEN	weighted by lone pair electronegativities
Novel EMAS Function weighted by sum of properties (24 descriptors)	EffectivePolarizability	weighted by effective atom polarizabilities
Novel EMAS Function weighted by product of properties (24 descriptors)	Vcharge	weighted by partial atomic charges accounting for alternate resonance forms
**Every Vector descriptor available with and without van der Waals surface area weighting**		

Table S2. Feature selection results with and without EMAS features. Novel EMAS features have been highlighted.

Control feature selection (without EMAS)		Novel feature selection (with EMAS)	
**Descriptor Type**	**Weight**	**Descriptor Type**	**Weight**
Radial Distribution Function	AtomIdentity [surface area scaled]	Radial Distribution Function	AtomIdentity [surface area scaled]
Radial Distribution Function	Vcharge	Radial Distribution Function	Vcharge
Radial Distribution Function	EffectivePolarizability [surface area scaled]	EMAS (product weight)	AtomIdentity [surface area scaled]
3D Autocorrelation	SigmaCharge	2D Autocorrelation	SigmaEN [surface area scaled]
Radial Distribution Function	LonePairEN	Radial Distribution Function	PiEN [surface area scaled]
2D Autocorrelation	SigmaEN	Scalar	HbondDonor
3D Autocorrelation	SigmaEN	EMAS (product weight)	SigmaEN [surface area scaled]
3D Autocorrelation	Vcharge [surface area scaled]	2D Autocorrelation	EffectivePolarizability [surface area scaled]
2D Autocorrelation	Vcharge [surface area scaled]	3D Autocorrelation	Vcharge [surface area scaled]
		Radial Distribution Function	PiEN
		3D Autocorrelation	SigmaCharge
		2D Autocorrelation	EffectivePolarizability
		EMAS (sum weight)	Vcharge [surface area scaled]
		EMAS (product weight)	Vcharge
		EMAS (sum weight)	TotalCharge
		Radial Distribution Function	EffectivePolarizability
		EMAS (sum weight)	LonePairEN
		EMAS (product weight)	PiEN [surface area scaled]
		3D Autocorrelation	PiEN [surface area scaled]
		Radial Distribution Function	SigmaCharge
